# Identification of Novel Pathways in Plant Lectin-Induced Cancer Cell Apoptosis

**DOI:** 10.3390/ijms17020228

**Published:** 2016-02-08

**Authors:** Zheng Shi, Rong Sun, Tian Yu, Rong Liu, Li-Jia Cheng, Jin-Ku Bao, Liang Zou, Yong Tang

**Affiliations:** 1 School of Basic Medical & Nursing School, Chengdu University, Chengdu 610106, China; drshiz1002@hotmail.com (Z.S.); yutian@edu.cn (T.Y.); liurongscu@126.com (R.L.); chenglijia007@hotmail.com (L.-J.C.); 2 School of Life Sciences and Key Laboratory of Bio-resources, Ministry of Education, Sichuan University, Chengdu 610064, China; sunrong198982@163.com (R.S.); baojinku@scu.edu.cn (J.-K.B.); 3 School of Acupuncture and Tuina, Chengdu University of Traditional Chinese Medicine, Chengdu 611137, China

**Keywords:** plant lectin, apoptosis, Naïve Bayesian model, novel pathways, cancer

## Abstract

Plant lectins have been investigated to elucidate their complicated mechanisms due to their remarkable anticancer activities. Although plant lectins seems promising as a potential anticancer agent for further preclinical and clinical uses, further research is still urgently needed and should include more focus on molecular mechanisms. Herein, a Naïve Bayesian model was developed to predict the protein-protein interaction (PPI), and thus construct the global human PPI network. Moreover, multiple sources of biological data, such as smallest shared biological process (SSBP), domain-domain interaction (DDI), gene co-expression profiles and cross-species interolog mapping were integrated to build the core apoptotic PPI network. In addition, we further modified it into a plant lectin-induced apoptotic cell death context. Then, we identified 22 apoptotic hub proteins in mesothelioma cells according to their different microarray expressions. Subsequently, we used combinational methods to predict microRNAs (miRNAs) which could negatively regulate the abovementioned hub proteins. Together, we demonstrated the ability of our Naïve Bayesian model-based network for identifying novel plant lectin-treated cancer cell apoptotic pathways. These findings may provide new clues concerning plant lectins as potential apoptotic inducers for cancer drug discovery.

## 1. Introduction

Plant lectins belong to a large family of carbohydrate-binding proteins with highly diverse non-immune origin. They contain at least one non-catalytic domain for selective recognizing and reversibly agglutinating cells [1]. In accordance with the molecular structures and evolutionary statuses, plant lectins can be further separated into 12 different families, namely, Amaranthin, *Agaricus bisporus* agglutinin, Cyanovirin, Chitinase-related agglutinin, *Euonymus europaeus* agglutinin, *Galanthus nivalis* agglutinin (GNA), Hevein, Jacalins, Lysine motif, proteins with legume lectin domains, Nictaba, and Ricin-B families [2].

Notably, plant lectins are well known to exert biological activities, such as anti-fungal, anti-viral activities, and particularity anti-tumor activities [3,4]. They can induce cancer cell death by targeting programmed cell death pathways and thus considered as a promising anticancer agent for future cancer therapy. And, programmed cell death, apoptosis and autophagy, play vital roles in homeostasis preservation, cellular differentiation, growth control, *etc.*, and might ultimately seal the fate of tumor cells.

Apoptosis, an organized genetically process of programmed events, is critical for the maintenance of tissue homeostasis and proper development [5]. And, the tightly regulated multi-step pathway of apoptosis is characterized by cell shrinkage, chromatin condensation, dynamics membrane blebbing and loss of adhesion [6]. Inactivation of pro-apoptotic proteins or up-regulation of anti-apoptotic proteins can lead to unchecked growth of cells and eventually leads to cancer. Previous findings have indicated that plant lectins possess anti-proliferative and apoptosis-inducing activities in a variety of cancer cell lines [7]. Therefore, an understanding of the molecular mechanisms of apoptosis supports the development of effective rational approaches to cancer treatment.

MicroRNAs, have emerged as another layer of gene regulation, and play a fundamental role in biological processes, including cell growth, death, development and differentiation, suggesting their potential roles in cancer. Hitherto, a growing number of publications show that some upregulated miRNAs, such as miRNA-21, miRNA-221, -222, and miRNA-272, -273 can be recognized as oncogenes while others that are down-regulated can be defined as tumor suppressor genes, such as miR-15a–miR-16-1 cluster, and miR-29. Dysregulation of miRNAs was reported to associate with various types of cancer [8,9]. Interestingly, Chinese mistletoe lectin-I (CMI) has been firstly reported to induce apoptotic cell death in colorectal cancer cells, by down-regulating miR-135a and miR-135b expression and up-regulating expression of their target gene adenomatous polyposis coli (APC), thereby, reducing activity of its downstream Wnt signaling [10]. Additionally, another report has demonstrated that nine autophagic hub proteins and 13 targeted miRNAs were identified in a plant lectin-induced cancer autophagic cell death context [11]. Wang and his colleagues predicted novel protein functional connections via a Naïve Bayesian model based on a human apoptotic protein-protein interaction (PPI) network, and several apoptotic hub proteins, such as *TP53*, *M3K3/5/8*, *CDK 2/6*, *TNFR16/9*, and *TGF-β* receptor 1/2 were further identified [12].

Hitherto, a bulk of studies have extensively been demonstrated that plant lectins can used as therapeutic agents, resulting cytotoxicity, apoptosis and inhibition of cancer cell proliferation. However, there is an additional drawback in the intrinsic molecular mechanisms of lectin, and thus there is a pressing demand for much more key information. Therefore, identification and validation of cancer-related novel pathways which are modulated by lectins should be of utmost importance.

With the increase of genome-wide data of genetic, functional and physical interactions, a robust mathematical model which would be suitable for integrating disparate type of data, seems to be imperative for inferring the apoptotic process. Of note, since the Naïve Bayesian model has been widely employed in constructing the global PPI networks in some model organisms, including yeast, fly, mouse and human, it is of great importance to integrate high-through data for predicting protein functional connections. Moreover, multiple analyses, including smallest shared biological process (SSBP), domain-domain interaction (DDI), gene co-expression profiles and cross-species interolog mapping were also utilized to make the data more accurate. However, to our best knowledge, there is no study to report novel pathways in plant lectin-induced apoptotic cell death context.

In the current study, we firstly took advantage of a Naïve Bayesian model to build the global human PPI network. Moreover, multiple sources of biological data were integrated to computationally construct a core global human PPI network which totally contained 109 proteins (3767 protein pairs), and we further modified it to a plant lectin-induced cancer cell apoptosis context. Twenty-two hub proteins associated with apoptosis and relevant miRNAs to interact with these hub proteins were identified by using microarray analysis. Consequently, the novel pathways in plant lectin-induced apoptosis which involved in miRNA regulations were finally identified based on the above-mentioned evidence. The identification of novel apoptotic pathways in plant lectin-induced cancer cell apoptosis may provide new evidence for plant lectin as antineoplastic agents in the near future.

## 2. Results and Discussion

### 2.1. Global Human Protein-Protein Interaction (PPI) Network Construction

The global human PPI network was built, covering almost all PPIs by using the Naïve Bayesian model. Several online PPI databases including 37,710 protein pairs from BioGRID, 14,892 from HomoMINT, 39,044 from Human Protein Reference Database (HPRD), 8044 from Biomolecular Object Network Databank (BOND), and 34,935 from IntAct were retrieved to construct the set of true-positive gene pairs. Then, 85,083 unique PPIs (13,128 proteins) were identified for the Gold Standard Positive (GSP) set, whereas 23,169,177 pairs are in Gold Standard Negative interaction (GSN) set. Thus, the ratio of GSP and GSN in our study reached 0.00367, which was similar with Rhodes’s study with the ratio of GSP and GSN of 0.00375, indicating that the number of negative set and positive set in our study was proper and reliable [13]. In addition, there are 25,620 protein pairs in the Standard Test set (STS) and 204,919,890 protein pairs in raw predicting data set, respectively. Based upon the two golden sets, multiple types of biological sources were integrated and the likelihood ratio (LR) was used as reliability of individual dataset for inferring the apoptotic PPI pairs [14]. Cutoff as 20 were used and the global PPI network including the positive set and the prediction set were constructed. We put the STS which contained 4818 unique proteins (12,809 protein pairs) into the model, caused the area under the receiver operating characteristic (ROC) curve (AUC) value. Then, the Naïve Bayesian model was launched. Consequently, the global human PPI network was identified.

### 2.2. Identification of the Core Apoptotic Pathways in Human PPI Network

Firstly, the apoptotic PPI network was built based on the abovementioned global human PPI network. Subsequently, hub proteins of the apoptotic pathway were identified on the basis of the following four standards. Firstly, proteins with high-degree generally play more significant roles in the process of apoptosis; we selected the proteins that were equal to or greater than 300 degrees as hub proteins which were identified as high degrees [15,16]; Secondly, the proteins which have 300 or 200 (the standard of novel hub proteins) apoptotic protein interactions were set as candidate hub proteins for further analysis; Thirdly, the hub proteins generally enrich in the “dense area” rather than “sparse area” in cancer [17,18,19]. A few conserved areas that could enrich more hub proteins in this sub-network were selected according to this description; Finally, significance analysis of microarrays (SAM) was performed to identify genes that are differentially expressed under apoptotic stress, and we indicated that those proteins which were extracted as hub proteins can be considered as different expression proteins [20,21,22]. Based on the aforementioned four golden standards, we identified a few of apoptotic hub proteins and their related signaling pathways to construct the predicted core apoptotic sub-network which totally consisted of 3767 protein pairs (shown in Table S1). More importantly, our standards of hub proteins are in good agreement with several previous studies, respectively or cooperatively, indicating that the combination of these standards which can be integrated into a more well-suited approach to help confirm apoptotic hub proteins in a specific context [22,23].

### 2.3. Identification of Plant Lectin-Induced Novel Apoptotic Pathways in Cancer

**Figure 1:**
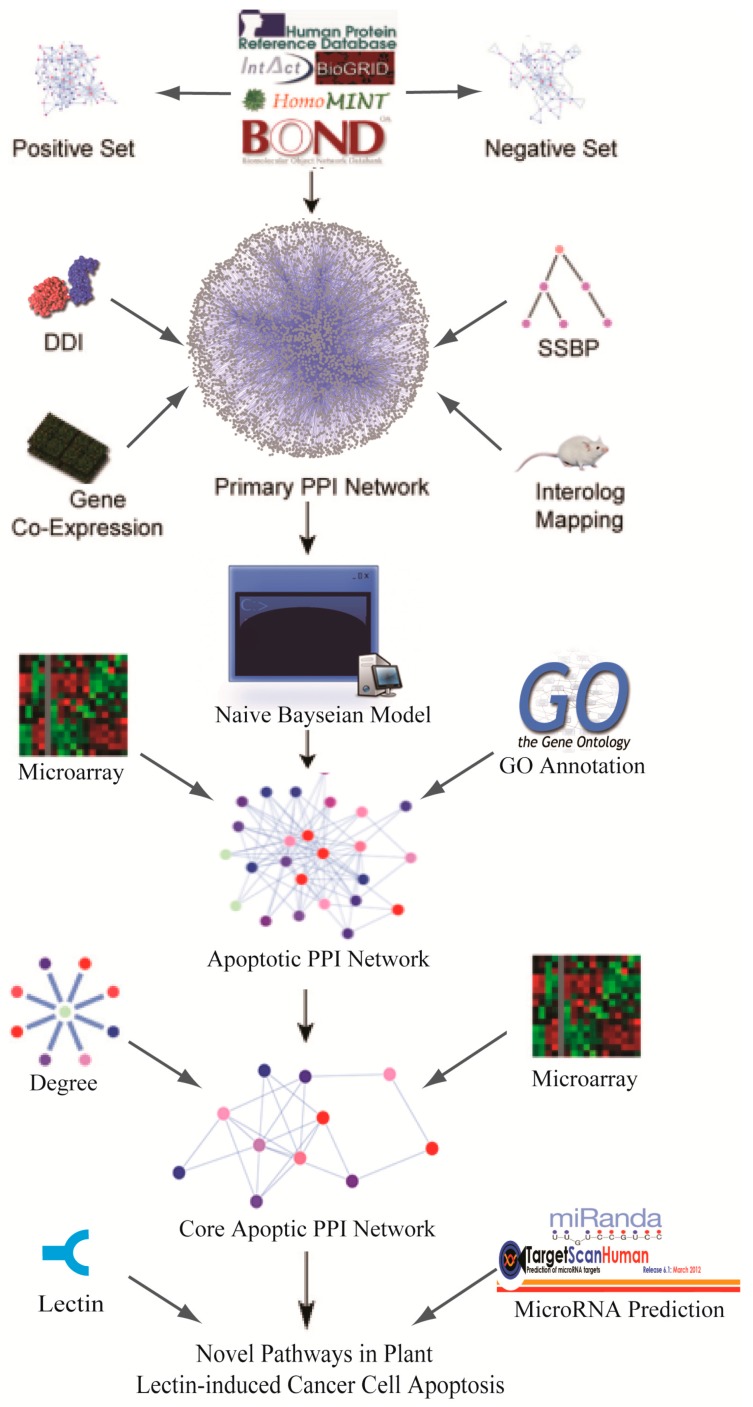
Schematic model of novel pathway prediction. DDI: domain-domain interaction; PPI: protein-protein interaction; GO: Gene Ontology.

**Figure 2:**
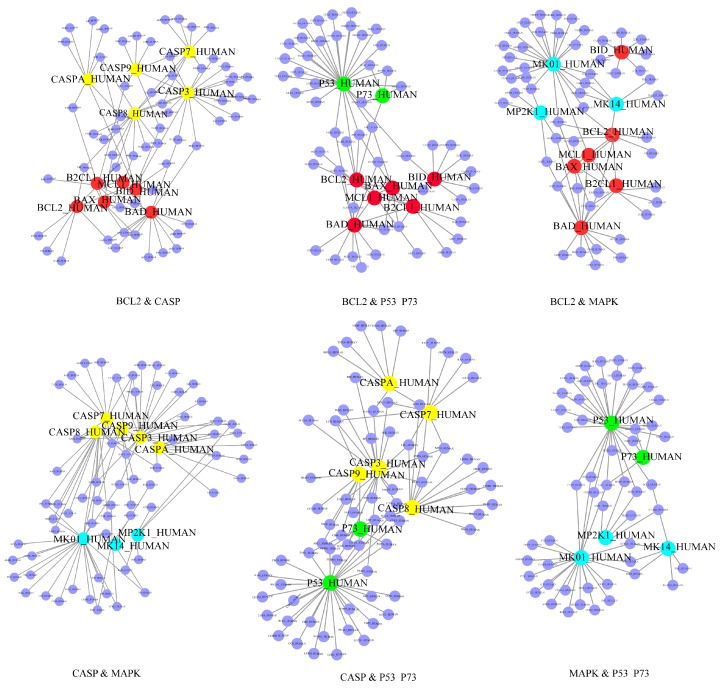
Several predicted PPI networks in apoptosis. Caspase family was marked in yellow balls; Bcl-2 family was mark in red balls; P53 family was marked in cyan balls; MAPKs family was marked in powderblue balls.

In the current study, the known apoptotic hub proteins in plant lectin-induced cancer cells were taken advantage of for further study (Table S2 [24]). A schematic model of this study is shown in Figure 1. Generally, hub proteins play more significant roles in this sub-network, so we extracted 30 hub proteins according to Table S1. Based upon the above analysis, we manually used a degree cutoff of 30 and finally obtained 27 proteins, indicating their significant roles in this sub-network. Regarding Table S2 [24], we extracted these 30 hub proteins which could be further classified into four families, which are the B-cell lymphoma-2 (Bcl-2) protein family, caspase protein family, mitogen-activated protein kinase (MAPK) protein family and p53 protein family. Moreover, family-family interactions were further analyzed (shown in Figure 2). Seen from this figure, we computationally predicted caspases and MAPKs that were both involved in the apoptotic network. Caspases such as caspase-3/caspase-2 can be regulated by Bcl-2 family which is well known apoptotic regulator. We also demonstrated that the caspase family and p53 showed a close relationship, which showed that the identified apoptotic PPI sub-network is relatively highly dependable.

Apoptosis is mainly responsible for the physiological removal of cells, which is regulated by numerous cellular signaling pathways and tightly related with cancer. There are two main apoptotic pathways, including the extrinsic or death receptor pathway and the intrinsic or mitochondrial pathway, and notably the caspase family is involved in these two different apoptotic pathways. Activation of downstream caspase-3 by both extrinsic and intrinsic pathways is responsible for execution of cancer cell death. MAPKs are participating converting cellular responses to a wide range of stimuli, including, osmotic stress, pro-inflammatory cytokines and mitogens. Bcl-2 family has either pro- or anti-apoptotic activities, and has been well known for its important function in the regulation of apoptosis and cellular response to anti-cancer therapy [25,26]. There are three transcription factors p53, p63 and p73 in the p53 family. P53 with various signaling pathways and inhibits growth as a tumor suppressor, and p53 also served as an upstream activator to control MAPK signaling pathway via the transcriptional activation of members of the dual specificity phosphatase family [27]. Elucidation of the functional interaction between altered p53 and MAPK signaling transduction pathways is critical for understanding how cell proliferation and survival are deregulated in cancer [28].

**Figure 3:**
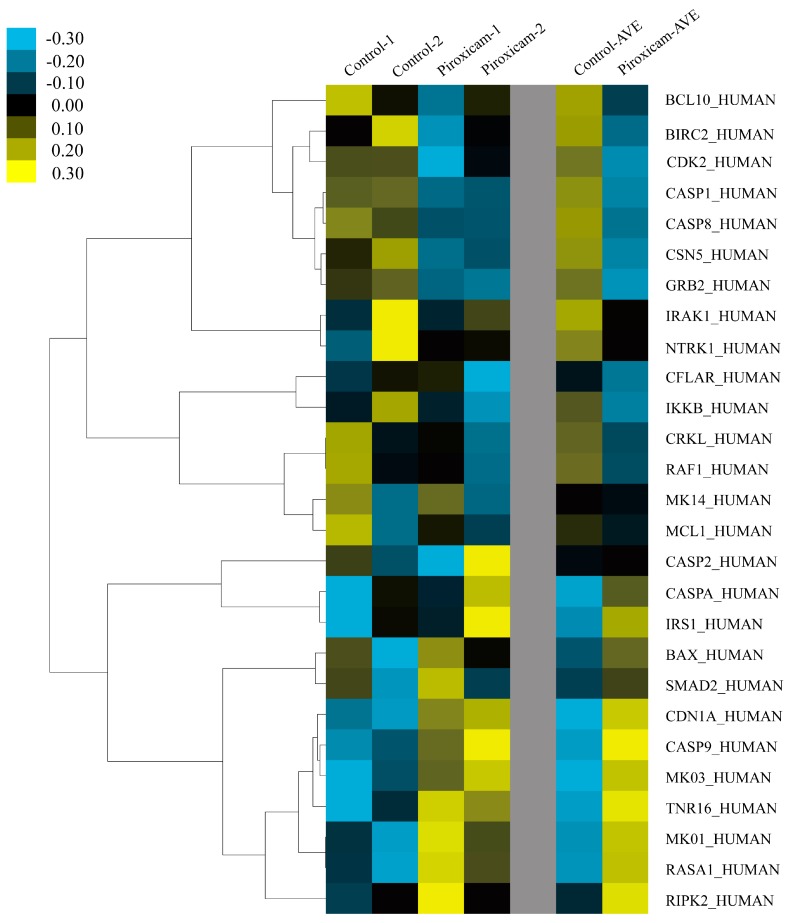
Microarray analyses of possible targets in mesothelioma.

**Figure 4:**
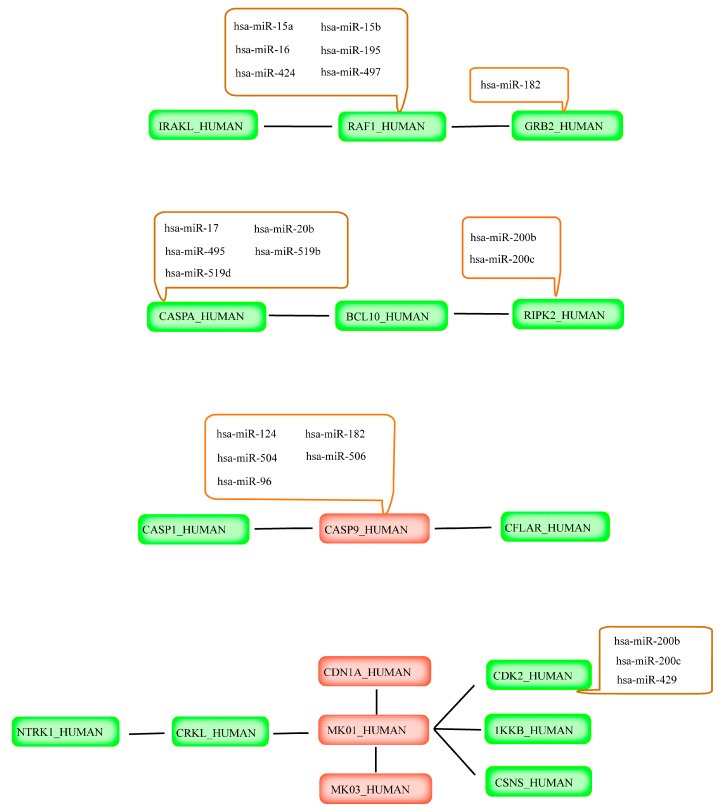
Novel pathways and relevant miRNA regulation in plant lectin-induced apoptosis context in mesothelioma cells. **Red**: Oncogene; **Green**: Tumor suppressor gene.

Subsequently, SAM analysis was utilized from piroxicam/cisplatin treated mesothelioma cell apoptosis, to declare differential gene expression between mesothelioma cells treated with piroxicam/cisplatin and the control group. Considering the gene co-expression profiles, we were able to identify the divergent expression hub proteins, suggesting their regulatory roles as potential targets in mesothelioma cells. Subsequently a differentially expressed gene signature was identified by SAM (Figure S1), and we finally recognized nine up-regulated and 13 down-regulation expression genes, which would be considered as important regulators in a plant lectin-induced apoptotic cell death (Figure 3). Based upon above descriptions, novel apoptotic pathways in plant lectin-induced in cancer were finally identified (Figure 4).

### 2.4. Prediction of miRNAs Targeting Apoptotic Hub Proteins

miRNAs are a class of negative gene regulators, and they have been reported to be involved in various physiological process, such as cellular proliferation, differentiation and apoptosis. Notably, miRNAs can act as either tumor suppressors or oncogenes; therefore, miRNAs could repress the expression of important cancer-related genes and have been already utilized for the diagnosis and treatment of cancer. More recently, increasing studies have indicated that some miRNAs are involved in apoptosis. In this work, we took advantage of Diana-MicroH, miRanda and TargetScan to predict the miRNAs targeting above-mentioned hub proteins. Then we integrated the results of the prediction into a consensus to find out which miRNAs specifically target the corresponding proteins. For example, caspase-9, 51 miRNAs through Diana-MicroH, 14 miRNAs by using miRanda and 156 miRNAs through TargetScan were successfully predicted. Subsequently, we integrated these miRNAs into consensus results. There are five miRNAs, including (*Homo sapiens*) hsa-miR-124, hsa-miR-182, hsa-miR-504, hsa -miR-506 and hsa-miR-96, and these five miRNAs may negatively regulate caspase-9-mediated signaling pathways. The prediction results of the miRNA are shown in Table 1 and Figure 4. In summary, these targeted miRNAs may regulate those hub proteins in plant lectin-induced apoptosis in cancer cells.

**Table 1:** Consensus results of predicted miRNA targeting receptors.

Gene Name	Protein Name	Consensus Results
*CASP9*	Caspase-9	hsa-miR-124
		hsa-miR-182
		hsa-miR-504
		hsa-miR-506
		hsa-miR-96
*RIPK2*	Receptor-interacting serine/threonine-protein kinase 2	hsa-miR-200b
		hsa-miR-200c
*GRB2*	Growth factor receptor-bound protein 2	hsa-miR-182
*CDK2*	Cyclin-dependent kinase 2	hsa-miR-200b
		hsa-miR-200c
		hsa-miR-429
*CASP8*	Caspase-8	hsa-miR-17
		hsa-miR-20b
		hsa-miR-495
		hsa-miR-519d
		hsa-miR-93
*RAF1*	Rapidly Accelerated Fibrosarcoma (RAF) proto-oncogene serine/threonine-protein kinase	hsa-miR-15a
		hsa-miR-15b
		hsa-miR-16
		hsa-miR-195
		hsa-miR-424
		hsa-miR-497

### 2.5. Plant Lectins as Promising Candidate for Drug Development

With the development of systems biology, carcinogenesis could be interpreted as the malfunction of perturbed protein functional interaction networks in a cell, and therefore, analyses of apoptosis-related network from a systems-level perspective is of great significance [29,30,31]. It is well known that cancer is a complex genetic disease resulting from mutations of tumor suppressor genes or oncogenes that would cause the alteration of signaling pathways. Accumulated evidence has revealed that Programmed cell death (PCD), including apoptosis and autophagy might serve as an effective cancer therapy since they could eliminate the damaged and deleterious cells [24]. Especially, apoptosis is known as one of the most important molecular mechanisms for tumor cell suicide.

Previous studies have indicated that mathematical models have been proposed to uncover several core pathways in apoptotic pathways, such as a nonlinear stochastic model and a large-scale literature-based Boolean model [22,32]. However, the PPI network is non-linear and complex and cannot depend on single biological evidence. Hence, it is necessary to represent the PPI network by using Naïve Bayesian model which could integrate disparate data into an advantageous platform [33]. Additionally, previous studies have proved that integrating multi biological data sources would provide more accuracy for identification of apoptotic hub protein/target. Generally, there is a necessity to represent the networks by further comprehensively analyzing the global PPI network in a specific context. Herein, we utilized a Naïve Bayesian model, which was well suited to integrated high-through biological data, including SSBP, DDI, gene co-expression profiles and cross-species interolog mapping, for possibly uncovering some core pathways in the apoptotic process. For further predicting novel apoptotic pathways implicated in cancer triggered by plant lectins, a sub-network was further extracted based on the proteins that were involved in lectin-induced apoptotic cell death. With the continuous exploration of molecular mechanisms of plant lectins, they have been extensively recognized as promising agents in human cancer. Intriguingly, recently studies have successfully demonstrated the ability of a Naïve Bayesian model-based network for recognizing the key double targets AMPK (Adenosine 5′-monophosphate (AMP)-activated protein kinase) and ZIPK (Zipper interacting protein kinase). Subsequently, they further provide the dual target small molecule, namely BL-AD008, as a potential new apoptosis modulating drug for cervical cancer therapy [34].

In plant lectin-induced apoptotic cell death context, 22 hub proteins and several relevant miRNA regulations in mesothelioma cells were eventually recognized due to their different microarray expressions. The novel apoptotic pathways and several miRNA regulations in plant lectin-induced context are shown in Figure 4. We found that CASP8_HUMAN was associated with BCL10_HUMAN and RIPK2_HUMAN, and some miRNAs, such as has-miR-200b/c negatively regulated RIPK2_HUMAN, were also involved in this pathway. Tumor suppressor CASP1_HUMAN and CFLAR_HUMAN was connected with oncogene CASP9_HUMAN, and several miRNAs, for example hsa-miR-124, hsa-miR-182, hsa-miR-504, hsa-miR-506 and has-miR-96 negatively regulated CASP9_HUMAN. All the above-mentioned novel pathways might pave a new road for plant lectins as potential apoptotic inducers in cancer drug discovery.

## 3. Materials and Methods

### 3.1. Retrieving of Functional Genomics Data

Experimentally verified protein interaction data was from five online databases, including Human Protein Reference Database (HPRD) [15,35], Biomolecular Object Network Databank (BOND) [15,36], IntAct [15,37], HomoMINT [15,38] and BioGRID [15,39]. Then, Gold Standard Positive interaction set (GSP) was constructed through the abovementioned five online databases. The Gold Standard Negative interaction set (GSN) was assigned by Gene Ontology (GO) Consortium according to protein pairs from the plasma membrane cellular component (6637 proteins) and from the nuclear cellular component (4138 proteins). Subsequently, 404 proteins were removed which were assign to those components, and there are also 5275 overlapping pairs with GSP. After this, 23,169,177 unique pairs were identified. Furthermore, we made the interactions from Database of Interacting Proteins [15,40] as data in Standard Test set (STS) to assess the mathematic model. For evaluation, we selected 12,809 interacting protein pairs from conformed by 4818 unique proteins into the model, resulting the area under AUC. The selected pairs were all from the data in GSP and GSN. Additionally, the ratio of GSP and GSN in STS reached 0.00367.

### 3.2. Integration of Several Biological Data Sources

#### 3.2.1. Smallest Shared Biological Process

Proteins interacting with each other are assumed to function in the same biological process, and proteins functioning in small specific processes are more likely to have interaction relationships compared to proteins functioning in larger general processes [13]. Functional similarities were quantified between two proteins in the following process. First, we identified all biological processes that two proteins shared. Then, the total number of other proteins which were involved in each of the processes was counted. Finally, we identified the shared biological process with the smallest count, because the smaller the count and the more specific the process, the greater functional similarity and greater possibility of interactions between two proteins.

#### 3.2.2. Domain–Domain Interaction

Numerous studies have elaborated that novel protein interactions could be calculated by distinguishing the pairs of domains enriched amongst observed interacting proteins, thereby, we downloaded DDI relationships from Pfam [15,41,42] to predict PPIs in GSN and GSP sets.

#### 3.2.3. Gene Co-Expression Profiles

Notably, co-expression of genes often bears similar gene expression patterns and such interaction might be an indicator of interactions between proteins. Microarray analysis of HeLa cells and primary human lung fibroblasts which treated with 2.5 mM DTT was used to assess the related genes co-expression level in apoptosis [20,43,44]. The co-expression level is computed by using Pearson Correlation Coefficient ρ.

#### 3.2.4. Cross-Species Interolog Mapping

The human orthologous proteins in model organism often keep the similar functions. If two proteins interact with each other in a certain model organism, the orthologs of the two proteins in human are likely to have interaction as well. Model organisms including *Caenorhabditis elegans* (4649), *Drosophila melanogaster* (5527), *Saccharomyces cerevisiae S288C* (2154), *Rattus norvegicus* (15,306) and *Musmusculus* (16,376), *Escherichia coli* K12 (541) were mapped to human protein pairs by gene orthologs to cluster into orthologous groups, which were defined in the Inparanoid database (Available online: http://inparanoid.sbc.su.se/cgi-bin/index.cgi). A number of pairwise ortholog groups between 17 whole genomes are formed to the Inparanoid eukaryotic ortholog database [45]. Moreover, the numbers after species indicated that the number of ortholog proteins between selected species and *Homo sapiens*. For each set, human protein pairs were classified as interolog or non-interolog, and the likelihood ratio were calculated, respectively. The LR cutoff indicates whether a pair of proteins interacting or not, and we filtered the candidate protein pairs through the Naïve Bayesian classifier by selecting the pairs whose LR exceed the cutoff.

#### 3.2.5. Naïve Bayesian Model Analysis

We developed a Naive Bayesian model to combine the abovementioned four data sources and to predict interactions in an integrated way [15,46]. According to the Bayesian theorem, the posterior odds given n evidence were computed as follows:

$$O_{posterior}=\frac{P(positive|E_{1},K\;,E_{n})}{P(negative|E_{1},K\;,E_{n})}$$

where positive means two proteins are functional related while negative means not. Herein, *P* indicates the probability that a pair of proteins could interact to each other. *E_1_*, *E_n_* indicate evidence 1 and evidence n, respectively. While *K* indicates symbol “...”. Then, we defined

$$LR_{\left(E_{1},K\;,E_{n}\right)\;}=\frac{P(E_{1},K\;,E_{n}|positive)}{P(E_{1},K\;,E_{n}|negtive)}$$

Oposterior = Oprior × *LR*. Since Naive Bayesian model supposes that each of the evidence is conditional independent, we can simplify *LR* as

$$LR_{\left(E_{1},K\;,E_{n}\right)}=\overset{n}{\underset{i=1}{\prod }}LR_{\left(E_{i}\right)}$$

As the prior odd is a constant, the predictive power or confidence degree was measured using the composite *LR* corresponding to a type of specific biological evidence. A cutoff of *LR* indicates whether protein pairs exert the functional linkages. Then, we filtered the initial networks through the Naïve Bayesian model by selecting the pairs with composite *LR* above the *LR* cutoff.

#### 3.2.6. Evaluation and Prediction of Naïve Bayesian Model

For different cut points, ROC curves can be used to elucidate a binary classifier system’s relationships between specificity and sensitivity [15,47], which can be represented equivalently by plotting the fraction of true positive rate (TPR) *versus* the fraction of false-positive rate (FPR). True positive (TP) indicates PPIs in Gold standard positive (GSP), and true negative indicates PPIs in Gold Standard Negative (GSN). Furthermore, false positive indicates those PPIs that belong to GSN while predicated as true apoptotic protein interactions by Naïve Bayesian model. On the contrary, false negtive indicates those PPIs that belong to GSP while predicated as non-apoptotic protein interactions by Naïve Bayesian mode. Sensitivity and specificity are calculated as sensitivity = TP/positives, and specificity = 1 − (FP/negatives), respectively. TP and FP are the number of true positives and false positives identified by a classifier, respectively; whereas positives and negatives are the total number of positives and negatives in a test. They reflect the ability of a classifier identifying true positives and false positives in a test.

The efficacy of the assessment system is presented by AUC, demonstrating that the larger the AUC is, the better the performance of the assessment system is. Therefore, for different classifiers, the performances are comparable by measuring the AUCs. Our Naïve Bayesian model was used to predict protein pairs from random matching and to select the protein pairs whose LR ratio is higher than the threshold which is regarded as the creditable results. The predicted core apoptotic PPI network was shown in Table S1.

#### 3.2.7. Identification of Hub Proteins

We defined whether or not an apoptotic protein is considered a hub protein, and followed gold standards, as follows. First, degree is defined as the number of edges per node, that is, the number of interacting partners. A node with high degree is called a network hub, which represents a protein with many interacting partner proteins [48,49]. Here, we used the cyto-Hubba (Hub Objects Analyzer; Taipei, Taiwan) plug-in to provide several topological analysis algorithms which included degree calculation for Cytoscape to explore important nodes/hubs in an interactome based on the previous framework, Hub object Analyzer (Hubba, available online: http://hub.iis.sinica.edu.tw) [50]. Secondly, the hub proteins should connect known apoptosis-related proteins in lectin-induced cancer cells [15]. Hence, the development of novel apoptosis-related targets was a worthwhile focus. This type of method was similar to a previous study that focused on identifying novel cancer-related genes [20].

In this study, we extracted known hub apoptotic proteins (Table S2 [24]) and their related pathways, based upon previous reports which noted that these proteins are involved in plant lectin-induced cancer cell apoptosis. Accordingly, these above-mentioned hub proteins can connect with one another to form the apoptotic PPI network, in which at least one protein can interact with the other, in lectin-treated cancer context.

#### 3.2.8. Microarray Analyses of Apoptotic Genes

In order to recognize genes whether co-expressed or not, we took advantage of microarray data (E-GEOD-22445), treated with piroxicam/cisplatin, to measure pair-wise co-expression gene in apoptosis in mesothelioma [48].

Microarray data were carried out three times repeatedly, in the control and experiment groups. The E-GEO database provided preliminary data that were further corrected and standardized. When the value of expression data was two, we used the average value, whereas when it was three or more, we adopted the median value.

Thus, data pre-processing would meet the standards by significance analysis of microarrays (SAM) [51,52], and the correspondingly identified probes were by ID from Uniprot database. As the preliminary experimental data did not make log_2_ processes relationship with the expression data, we set the log_2_ conduct and *T*-test in statistic model, and used “Two class (Unpaired)” model in SAM for analyses of different gene expressions.

Due to the above analyses, delta value in SAM plot controller was properly adjusted to limit the false discovery rate (FDR) within 5% and showed all the different expression genes (up-regulation or down-regulation). Firstly, we made all data undergo log_2_ alternation then normalization, and used the correlation and complete linkage models to cluster the data (Prior to this analysis, we extracted these gene expression data). Finally, T-statistic was used to test the gene expression signal, and all resulted differential genes (upregulated and downregulated) were listed in the SAM output sheet for further analyses. Moreover, Cluster 3.0 (Open source clustering software (de Hoon MJ *et al.*; Tokyo, Japan) [53] and Java Treeview 1.0 (Java Treeview—extensible visualization of microarray data; Saldanha, Palo Alto, CA, USA) [54] were also used to analyze and delineate the clustering of all significant genes.

#### 3.2.9. Targeted miRNA Prediction and Classification

It is reported that a combination of methods might provide a better understanding of complex regulatory mechanisms that involves miRNAs [55]. Therefore, we took advantage of computational predictions by sources of three algorithmically different methods, namely, TargetScan (stringent seed pairing, site number, site type, site context, option of ranking by likelihood of preferential conservation rather than site context) [56], MiRanda (moderately stringent seed pairing, site number, pairing to most of the miRNA) [57] and Diana-MicroT (hybridization energy threshold rules), respectively [58].

## 4. Conclusions

In summary, the global human PPI network was computationally constructed by using a Naïve Bayesian model. Subsequently, the core apoptotic PPI networks were built using several biological datasets, including SSBP, DDI, gene co-expression profiles and cross-species interolog mapping. Additionally, we further modified these networks into a plant lectin-induced apoptosis context. In total, 22 hub proteins including the caspase family, cyclin-dependent kinases (CDKs), as well as MAPK-activated protein kinases (MKs), and targeted miRNAs were successfully observed in this sub-network. The core apoptotic sub-network and relevant miRNAs might provide new molecule mechanism for apoptosis involving identification of core apoptotic pathways. Taken together, novel pathways were identified which might shed new light on the development of plant lectins as potent apoptotic inducers for future cancer drug development.

## Supplementary Materials

Supplementary materials can be found at http://www.mdpi.com/1422-0067/17/2/228/s1.
