# Supplementary material for: Identification of Novel Pathways in Plant Lectin-Induced Cancer Cell Apoptosis
Source: Int J Mol Sci. 2016 Feb 8;17(2):228. doi: 10.3390/ijms17020228 (PMC4783960; doi:10.3390/ijms17020228)
Supplement: Supplementary file 1 [file ijms-17-00228-s001.pdf]

SupplementaryMaterials: Identification of Novel Pathways in Plant Lectin-Induced Cancer Cell Apoptosis

Zheng Shi, Rong Sun, Tian Yu, Rong Liu, Li-Jia Cheng, Jin-Ku Bao, Liang Zou and Yong Tang

Table S1. The predicted core apoptotic protein-protein interaction (PPI) network.

Protein 1	Protein 2
ABL1_HUMAN	1433B_HUMAN
M3K5_HUMAN	1433B_HUMAN
MYC_HUMAN	1433B_HUMAN
RAF1_HUMAN	1433B_HUMAN
ABL1_HUMAN	1433E_HUMAN
CASP3_HUMAN	1433E_HUMAN
EGFR_HUMAN	1433E_HUMAN
MYC_HUMAN	1433E_HUMAN
BAD_HUMAN	1433F_HUMAN
M3K5_HUMAN	1433F_HUMAN
BAD_HUMAN	1433G_HUMAN
ABL1_HUMAN	1433T_HUMAN
BAD_HUMAN	1433T_HUMAN
BAX_HUMAN	1433T_HUMAN
EGFR_HUMAN	1433T_HUMAN
M3K5_HUMAN	1433T_HUMAN
MYC_HUMAN	1433T_HUMAN
A4_HUMAN	1433Z_HUMAN
AKT1_HUMAN	1433Z_HUMAN
ARRB1_HUMAN	1433Z_HUMAN
CBL_HUMAN	1433Z_HUMAN
EGFR_HUMAN	1433Z_HUMAN
M3K5_HUMAN	1433Z_HUMAN
MK08_HUMAN	1433Z_HUMAN
MP2K1_HUMAN	1433Z_HUMAN
NEMO_HUMAN	1433Z_HUMAN
NR4A1_HUMAN	1433Z_HUMAN
CUL1_HUMAN	2AAA_HUMAN
NFKB2_HUMAN	2AAA_HUMAN
PP2AA_HUMAN	2AAA_HUMAN
P53_HUMAN	2ABB_HUMAN
SMAD2_HUMAN	2ABB_HUMAN
SMAD3_HUMAN	2ABB_HUMAN
SMUF1_HUMAN	2ABB_HUMAN
VHL_HUMAN	2ABB_HUMAN
CASP4_HUMAN	A4_HUMAN
CASP6_HUMAN	A4_HUMAN
G3P_HUMAN	A4_HUMAN
ATM_HUMAN	ABL1_HUMAN
CRK_HUMAN	ABL1_HUMAN
2ABB_HUMAN	ACTB_HUMAN
A4_HUMAN	ACTB_HUMAN
SMAD3_HUMAN	ACTB_HUMAN
1433Z_HUMAN	ACTG_HUMAN
IKBA_HUMAN	ACTG_HUMAN
IKKB_HUMAN	ACTG_HUMAN
IKKE_HUMAN	ACTG_HUMAN

Table S1.*Cont.*

Protein 1	Protein 2
M3K1_HUMAN	ACTG_HUMAN
MCL1_HUMAN	ACTG_HUMAN
MYC_HUMAN	ACTG_HUMAN
NEMO_HUMAN	ACTG_HUMAN
NFKB1_HUMAN	ACTG_HUMAN
RIPK2_HUMAN	ACTG_HUMAN
SMAD3_HUMAN	ACTG_HUMAN
TF65_HUMAN	ACTG_HUMAN
TNR1A_HUMAN	ACTG_HUMAN
TNR1B_HUMAN	ACTG_HUMAN
TRADD_HUMAN	ACTG_HUMAN
TRAF1_HUMAN	ACTG_HUMAN
TRAF2_HUMAN	ACTG_HUMAN
TRAF6_HUMAN	ACTG_HUMAN
MDM2_HUMAN	AKT1_HUMAN
NARG2_HUMAN	AKT1_HUMAN
TAU_HUMAN	AKT1_HUMAN
BRCA1_HUMAN	ANDR_HUMAN
CDK1_HUMAN	ANDR_HUMAN
CEBPB_HUMAN	ANDR_HUMAN
GSK3B_HUMAN	ANDR_HUMAN
HSP71_HUMAN	ANDR_HUMAN
MK01_HUMAN	ANDR_HUMAN
MK01_HUMAN	ARRB1_HUMAN
MK01_HUMAN	ARRB2_HUMAN
RAF1_HUMAN	ARRB2_HUMAN
KAT5_HUMAN	ATM_HUMAN
NEMO_HUMAN	ATM_HUMAN
NEMO_HUMAN	ATR_HUMAN
ABL1_HUMAN	B2CL1_HUMAN
BAK_HUMAN	B2CL1_HUMAN
BAX_HUMAN	B2CL1_HUMAN
BCL2_HUMAN	B2CL1_HUMAN
MK08_HUMAN	B2CL1_HUMAN
1433Z_HUMAN	BAD_HUMAN
B2CL1_HUMAN	BAD_HUMAN
KAPCA_HUMAN	BAD_HUMAN
MCL1_HUMAN	BAD_HUMAN
PKN1_HUMAN	BAD_HUMAN
PP1A_HUMAN	BAD_HUMAN
RAF1_HUMAN	BAD_HUMAN
B2CL1_HUMAN	BAK_HUMAN
MCL1_HUMAN	BAK_HUMAN
B2CL1_HUMAN	BAX_HUMAN
ORN_HUMAN	BAX_HUMAN
PP1A_HUMAN	BAX_HUMAN
BAK_HUMAN	BCL10_HUMAN
BAX_HUMAN	BCL10_HUMAN
CASP1_HUMAN	BCL10_HUMAN
CASP2_HUMAN	BCL10_HUMAN
CASP8_HUMAN	BCL10_HUMAN
CDK2_HUMAN	BCL10_HUMAN
CFLAR_HUMAN	BCL10_HUMAN
NEMO_HUMAN	BCL10_HUMAN
NOD1_HUMAN	BCL10_HUMAN
B2CL1_HUMAN	BCL2_HUMAN

Table S1. *Cont.*

Protein 1	Protein 2
BAX_HUMAN	BCL2_HUMAN
MCL1_HUMAN	BCL2_HUMAN
P53_HUMAN	BCL2_HUMAN
RASH_HUMAN	BCL2_HUMAN
NCOR1_HUMAN	BCL6_HUMAN
ATR_HUMAN	BID_HUMAN
CASP1_HUMAN	BID_HUMAN
CASP2_HUMAN	BID_HUMAN
CASP3_HUMAN	BID_HUMAN
CSK21_HUMAN	BID_HUMAN
EZRI_HUMAN	BID_HUMAN
NEMO_HUMAN	BIRC2_HUMAN
RIP_HUMAN	BIRC2_HUMAN
UBB_HUMAN	BIRC2_HUMAN
TF65_HUMAN	BTK_HUMAN
TNFL6_HUMAN	BTK_HUMAN
1433Z_HUMAN	CALM_HUMAN
2ABB_HUMAN	CALM_HUMAN
IKBA_HUMAN	CALM_HUMAN
M3K7_HUMAN	CALM_HUMAN
NEMO_HUMAN	CALM_HUMAN
TF65_HUMAN	CALM_HUMAN
TNR1A_HUMAN	CALM_HUMAN
TNR1B_HUMAN	CALM_HUMAN
TNR6_HUMAN	CALM_HUMAN
TRADD_HUMAN	CALM_HUMAN
TRAF2_HUMAN	CALM_HUMAN
TRAF6_HUMAN	CALM_HUMAN
VHL_HUMAN	CALM_HUMAN
ANDR_HUMAN	CASP1_HUMAN
CASP2_HUMAN	CASP1_HUMAN
CASP9_HUMAN	CASP1_HUMAN
CFLAR_HUMAN	CASP1_HUMAN
ANDR_HUMAN	CASP3_HUMAN
SLK_HUMAN	CASP3_HUMAN
BCL10_HUMAN	CASP4_HUMAN
CFLAR_HUMAN	CASP4_HUMAN
RIPK2_HUMAN	CASP4_HUMAN
ANDR_HUMAN	CASP7_HUMAN
GRP78_HUMAN	CASP7_HUMAN
IKKE_HUMAN	CASP7_HUMAN
LYN_HUMAN	CASP7_HUMAN
ANDR_HUMAN	CASP8_HUMAN
EZRI_HUMAN	CASP8_HUMAN
IKKA_HUMAN	CASP8_HUMAN
IKKB_HUMAN	CASP8_HUMAN
NEMO_HUMAN	CASP8_HUMAN
ABL1_HUMAN	CASP9_HUMAN
CASP3_HUMAN	CASP9_HUMAN
CFLAR_HUMAN	CASP9_HUMAN
LYN_HUMAN	CASP9_HUMAN
XIAP_HUMAN	CASP9_HUMAN
BID_HUMAN	CASPA_HUMAN
EZRI_HUMAN	CASPA_HUMAN
MK08_HUMAN	CASPA_HUMAN

Table S1.*Cont.*

Protein 1	Protein 2
RHOA_HUMAN	CASPA_HUMAN
TNR1B_HUMAN	CAV1_HUMAN
CEBPB_HUMAN	CBP_HUMAN
CDK1_HUMAN	CCNB1_HUMAN
CUL1_HUMAN	CCNB1_HUMAN
MDM2_HUMAN	CD2A2_HUMAN
P53_HUMAN	CD2A2_HUMAN
2ABB_HUMAN	CDC42_HUMAN
CASP7_HUMAN	CDC42_HUMAN
ATR_HUMAN	CDK1_HUMAN
CCNB1_HUMAN	CDK1_HUMAN
KAT5_HUMAN	CDK1_HUMAN
NARG2_HUMAN	CDK1_HUMAN
ORN_HUMAN	CDK1_HUMAN
PCNA_HUMAN	CDK1_HUMAN
PIN1_HUMAN	CDK1_HUMAN
UBC_HUMAN	CDK1_HUMAN
ZBT16_HUMAN	CDK1_HUMAN
CDN1A_HUMAN	CDK2_HUMAN
MK01_HUMAN	CDK2_HUMAN
VHL_HUMAN	CDK2_HUMAN
HDAC4_HUMAN	CDK5_HUMAN
NARG2_HUMAN	CDK5_HUMAN
P53_HUMAN	CDK5_HUMAN
CASP3_HUMAN	CDN1A_HUMAN
CBP_HUMAN	CDN1A_HUMAN
CDK2_HUMAN	CDN1A_HUMAN
ESR1_HUMAN	CDN1A_HUMAN
MK01_HUMAN	CDN1A_HUMAN
PCNA_HUMAN	CDN1A_HUMAN
STAT3_HUMAN	CDN1A_HUMAN
1433E_HUMAN	CEBPB_HUMAN
EGFR_HUMAN	CEBPB_HUMAN
MK01_HUMAN	CEBPB_HUMAN
P53_HUMAN	CEBPB_HUMAN
SP1_HUMAN	CEBPB_HUMAN
STA5A_HUMAN	CEBPB_HUMAN
TGFR2_HUMAN	CEBPB_HUMAN
DAXX_HUMAN	CFLAR_HUMAN
FADD_HUMAN	CFLAR_HUMAN
TRAF2_HUMAN	CFLAR_HUMAN
UBC_HUMAN	CFLAR_HUMAN
KPCA_HUMAN	CFTR_HUMAN
1433Z_HUMAN	CH60_HUMAN
2ABB_HUMAN	CH60_HUMAN
ACTB_HUMAN	CH60_HUMAN
CASP9_HUMAN	CH60_HUMAN
CFTR_HUMAN	CH60_HUMAN
HSP71_HUMAN	CH60_HUMAN
KAPCA_HUMAN	CH60_HUMAN
PIN1_HUMAN	CH60_HUMAN
VHL_HUMAN	CRCM_HUMAN
TNFL6_HUMAN	CRK_HUMAN
HDAC3_HUMAN	CSK21_HUMAN
JUN_HUMAN	CSK21_HUMAN

Table S1. *Cont.*

Protein 1	Protein 2
PML_HUMAN	CSK21_HUMAN
MK01_HUMAN	CSN5_HUMAN
VHL_HUMAN	CSN5_HUMAN
ABL1_HUMAN	CTNB1_HUMAN
CASP8_HUMAN	CTNB1_HUMAN
CSK21_HUMAN	CTNB1_HUMAN
FYN_HUMAN	CTNB1_HUMAN
MUC1_HUMAN	CTNB1_HUMAN
PIN1_HUMAN	CTNB1_HUMAN
SLK_HUMAN	CTNB1_HUMAN
CRCM_HUMAN	CUL1_HUMAN
CSN5_HUMAN	CUL1_HUMAN
IKKA_HUMAN	CUL1_HUMAN
PP1A_HUMAN	CUL1_HUMAN
UBC_HUMAN	CUL1_HUMAN
ANDR_HUMAN	DAXX_HUMAN
TNR6_HUMAN	DAXX_HUMAN
1433Z_HUMAN	EF1A1_HUMAN
2ABB_HUMAN	EF1A1_HUMAN
EGFR_HUMAN	EF1A1_HUMAN
NR4A1_HUMAN	EF1A1_HUMAN
TNR6_HUMAN	EF1A1_HUMAN
CASP1_HUMAN	EGFR_HUMAN
GRB2_HUMAN	EGFR_HUMAN
MUC1_HUMAN	EGFR_HUMAN
AKT1_HUMAN	EP300_HUMAN
CDK2_HUMAN	EP300_HUMAN
CEBPB_HUMAN	EP300_HUMAN
ESR1_HUMAN	EP300_HUMAN
HIF1A_HUMAN	EP300_HUMAN
KPCD_HUMAN	EP300_HUMAN
MP2K1_HUMAN	EP300_HUMAN
MUC1_HUMAN	EP300_HUMAN
PAG1_HUMAN	EP300_HUMAN
ESR1_HUMAN	ERBB2_HUMAN
MUC1_HUMAN	ERBB2_HUMAN
UBB_HUMAN	ERBB2_HUMAN
MUC1_HUMAN	ERBB3_HUMAN
CEBPB_HUMAN	ESR1_HUMAN
GRB2_HUMAN	ESR1_HUMAN
IKKA_HUMAN	ESR1_HUMAN
KAT5_HUMAN	ESR1_HUMAN
M3K1_HUMAN	ESR1_HUMAN
MK14_HUMAN	ESR1_HUMAN
NARG2_HUMAN	ESR1_HUMAN
PKN1_HUMAN	ESR1_HUMAN
PTN6_HUMAN	ESR1_HUMAN
SHC1_HUMAN	ESR1_HUMAN
SRC_HUMAN	ESR1_HUMAN
BAD_HUMAN	EWS_HUMAN
1433Z_HUMAN	EZRI_HUMAN
CDK5_HUMAN	EZRI_HUMAN
IKKE_HUMAN	EZRI_HUMAN
RIPK2_HUMAN	EZRI_HUMAN
TNR6_HUMAN	EZRI_HUMAN

Table S1.*Cont.*

Protein 1	Protein 2
VHL_HUMAN	EZRI_HUMAN
CFLAR_HUMAN	FADD_HUMAN
EZRI_HUMAN	FADD_HUMAN
RIP_HUMAN	FADD_HUMAN
TNR1A_HUMAN	FADD_HUMAN
TNR6_HUMAN	FADD_HUMAN
TRADD_HUMAN	FADD_HUMAN
TNR1A_HUMAN	FAK1_HUMAN
ESR1_HUMAN	FHL2_HUMAN
PSN2_HUMAN	FHL2_HUMAN
TNFL6_HUMAN	FINC_HUMAN
VHL_HUMAN	FINC_HUMAN
1433Z_HUMAN	FLNA_HUMAN
NEMO_HUMAN	FLNA_HUMAN
PSN1_HUMAN	FLNA_HUMAN
RAC1_HUMAN	FLNA_HUMAN
TNR1B_HUMAN	FLNA_HUMAN
TRAF2_HUMAN	FLNA_HUMAN
HSP71_HUMAN	FOS_HUMAN
JUN_HUMAN	FOS_HUMAN
CASP3_HUMAN	FYN_HUMAN
1433Z_HUMAN	G3P_HUMAN
EGFR_HUMAN	G3P_HUMAN
MCL1_HUMAN	G3P_HUMAN
RIPK2_HUMAN	G3P_HUMAN
TNR1B_HUMAN	G3P_HUMAN
TRAF1_HUMAN	G3P_HUMAN
HSP71_HUMAN	GCR_HUMAN
MK01_HUMAN	GCR_HUMAN
MK08_HUMAN	GCR_HUMAN
P53_HUMAN	GCR_HUMAN
PRKDC_HUMAN	GCR_HUMAN
SMAD3_HUMAN	GCR_HUMAN
STA5A_HUMAN	GCR_HUMAN
STA5B_HUMAN	GCR_HUMAN
ZBT16_HUMAN	GCR_HUMAN
A4_HUMAN	GRB2_HUMAN
BAD_HUMAN	GRB2_HUMAN
RAF1_HUMAN	GRB2_HUMAN
SMAD3_HUMAN	GRB2_HUMAN
TNR16_HUMAN	GRB2_HUMAN
1433G_HUMAN	GRP75_HUMAN
2ABB_HUMAN	GRP75_HUMAN
ARRB1_HUMAN	GRP75_HUMAN
CFTR_HUMAN	GRP75_HUMAN
EGFR_HUMAN	GRP75_HUMAN
GRP78_HUMAN	GRP75_HUMAN
H2AX_HUMAN	GRP75_HUMAN
HS90B_HUMAN	GRP75_HUMAN
IKKE_HUMAN	GRP75_HUMAN
M3K1_HUMAN	GRP75_HUMAN
M3K14_HUMAN	GRP75_HUMAN
M3K3_HUMAN	GRP75_HUMAN
M3K7_HUMAN	GRP75_HUMAN
NEMO_HUMAN	GRP75_HUMAN

Table S1. *Cont.*

Protein 1	Protein 2
NFKB2_HUMAN	GRP75_HUMAN
RIPK1_HUMAN	GRP75_HUMAN
TNR1A_HUMAN	GRP75_HUMAN
TNR1B_HUMAN	GRP75_HUMAN
TRAF1_HUMAN	GRP75_HUMAN
TRAF2_HUMAN	GRP75_HUMAN
2ABB_HUMAN	GRP78_HUMAN
M3K1_HUMAN	GRP78_HUMAN
M3K14_HUMAN	GRP78_HUMAN
M3K3_HUMAN	GRP78_HUMAN
NEMO_HUMAN	GRP78_HUMAN
NFKB2_HUMAN	GRP78_HUMAN
RAF1_HUMAN	GRP78_HUMAN
RUVB2_HUMAN	GRP78_HUMAN
TNR1B_HUMAN	GRP78_HUMAN
AKT1_HUMAN	GSK3B_HUMAN
MYC_HUMAN	GSK3B_HUMAN
NEMO_HUMAN	GSK3B_HUMAN
PTN1_HUMAN	GSK3B_HUMAN
ATM_HUMAN	H2AX_HUMAN
BRCA1_HUMAN	H4_HUMAN
HDAC1_HUMAN	H4_HUMAN
M3K1_HUMAN	H4_HUMAN
M3K7_HUMAN	H4_HUMAN
TNR1A_HUMAN	H4_HUMAN
BAD_HUMAN	HCLS1_HUMAN
CASP3_HUMAN	HCLS1_HUMAN
CDN1A_HUMAN	HDAC1_HUMAN
CSK21_HUMAN	HDAC1_HUMAN
ESR1_HUMAN	HDAC1_HUMAN
GCR_HUMAN	HDAC1_HUMAN
NARG2_HUMAN	HDAC1_HUMAN
UBC9_HUMAN	HDAC1_HUMAN
HDAC3_HUMAN	HDAC2_HUMAN
IKKE_HUMAN	HDAC2_HUMAN
ANDR_HUMAN	HDAC3_HUMAN
UBC_HUMAN	HDAC3_HUMAN
1433Z_HUMAN	HDAC4_HUMAN
EP300_HUMAN	HIF1A_HUMAN
VHL_HUMAN	HIF1A_HUMAN
1433Z_HUMAN	HS71L_HUMAN
HSP71_HUMAN	HS71L_HUMAN
IKBA_HUMAN	HS71L_HUMAN
IKKB_HUMAN	HS71L_HUMAN
M3K1_HUMAN	HS71L_HUMAN
M3K7_HUMAN	HS71L_HUMAN
NEMO_HUMAN	HS71L_HUMAN
NFKB1_HUMAN	HS71L_HUMAN
P53_HUMAN	HS71L_HUMAN
RIPK1_HUMAN	HS71L_HUMAN
RIPK2_HUMAN	HS71L_HUMAN
TF65_HUMAN	HS71L_HUMAN
TNR1A_HUMAN	HS71L_HUMAN
TNR1B_HUMAN	HS71L_HUMAN
TRADD_HUMAN	HS71L_HUMAN

Table S1. *Cont.*

Protein 1	Protein 2
TRAF1_HUMAN	HS71L_HUMAN
TRAF2_HUMAN	HS71L_HUMAN
TRAF6_HUMAN	HS71L_HUMAN
1433Z_HUMAN	HS90A_HUMAN
CH60_HUMAN	HS90A_HUMAN
CUL1_HUMAN	HS90A_HUMAN
M3K7_HUMAN	HS90A_HUMAN
NOD1_HUMAN	HS90A_HUMAN
RAF1_HUMAN	HS90A_HUMAN
RIPK1_HUMAN	HS90A_HUMAN
TNR1A_HUMAN	HS90A_HUMAN
TRADD_HUMAN	HS90A_HUMAN
1433Z_HUMAN	HS90B_HUMAN
CUL1_HUMAN	HS90B_HUMAN
NEMO_HUMAN	HS90B_HUMAN
RAF1_HUMAN	HS90B_HUMAN
RIPK1_HUMAN	HS90B_HUMAN
TF65_HUMAN	HS90B_HUMAN
TRADD_HUMAN	HS90B_HUMAN
1433G_HUMAN	HSP71_HUMAN
1433T_HUMAN	HSP71_HUMAN
2ABB_HUMAN	HSP71_HUMAN
CFTR_HUMAN	HSP71_HUMAN
CSN5_HUMAN	HSP71_HUMAN
GRP75_HUMAN	HSP71_HUMAN
GRP78_HUMAN	HSP71_HUMAN
H2AX_HUMAN	HSP71_HUMAN
KPCA_HUMAN	HSP71_HUMAN
NEMO_HUMAN	HSP71_HUMAN
NOD1_HUMAN	HSP71_HUMAN
1433Z_HUMAN	HSP74_HUMAN
GRP78_HUMAN	HSP74_HUMAN
HDAC1_HUMAN	HSP74_HUMAN
HDAC3_HUMAN	HSP74_HUMAN
IKBA_HUMAN	HSP74_HUMAN
RAF1_HUMAN	HSP74_HUMAN
1433Z_HUMAN	HSP7C_HUMAN
2ABB_HUMAN	HSP7C_HUMAN
A4_HUMAN	HSP7C_HUMAN
EGFR_HUMAN	HSP7C_HUMAN
HDAC3_HUMAN	HSP7C_HUMAN
IKKB_HUMAN	HSP7C_HUMAN
JAK2_HUMAN	HSP7C_HUMAN
M3K1_HUMAN	HSP7C_HUMAN
M3K7_HUMAN	HSP7C_HUMAN
NFKB1_HUMAN	HSP7C_HUMAN
RAF1_HUMAN	HSP7C_HUMAN
RIPK1_HUMAN	HSP7C_HUMAN
RIPK2_HUMAN	HSP7C_HUMAN
TNR1B_HUMAN	HSP7C_HUMAN
TRADD_HUMAN	HSP7C_HUMAN
TRAF1_HUMAN	HSP7C_HUMAN
TRAF2_HUMAN	HSP7C_HUMAN
TRAF6_HUMAN	HSP7C_HUMAN
CFTR_HUMAN	HSPB1_HUMAN
EGFR_HUMAN	HSPB1_HUMAN

Table S1.*Cont.*

Protein 1	Protein 2
GRB2_HUMAN	HSPB1_HUMAN
TRAF2_HUMAN	HSPB1_HUMAN
CUL1_HUMAN	IKBA_HUMAN
IKKA_HUMAN	IKBA_HUMAN
NCOR2_HUMAN	IKBA_HUMAN
NFKB1_HUMAN	IKBA_HUMAN
NFKB2_HUMAN	IKBA_HUMAN
PTN1_HUMAN	IKBA_HUMAN
SUMO1_HUMAN	IKBA_HUMAN
TBB4_HUMAN	IKBA_HUMAN
TERA_HUMAN	IKBA_HUMAN
UBC_HUMAN	IKBA_HUMAN
CTNB1_HUMAN	IKKA_HUMAN
IKKB_HUMAN	IKKA_HUMAN
PRKDC_HUMAN	IKKA_HUMAN
SMAD3_HUMAN	IKKA_HUMAN
TF65_HUMAN	IKKA_HUMAN
CBP_HUMAN	IKKB_HUMAN
CTNB1_HUMAN	IKKB_HUMAN
HS90A_HUMAN	IKKB_HUMAN
IKBA_HUMAN	IKKB_HUMAN
MK01_HUMAN	IKKB_HUMAN
NEMO_HUMAN	IKKB_HUMAN
PRKDC_HUMAN	IKKB_HUMAN
TRAF2_HUMAN	IKKB_HUMAN
UBB_HUMAN	IKKB_HUMAN
HS90A_HUMAN	IKKE_HUMAN
1433Z_HUMAN	IMB1_HUMAN
EGFR_HUMAN	IMB1_HUMAN
TNR1B_HUMAN	IMB1_HUMAN
TRADD_HUMAN	IMB1_HUMAN
RAF1_HUMAN	IRAK1_HUMAN
UBC_HUMAN	IRAK1_HUMAN
1433Z_HUMAN	IRS1_HUMAN
RASA1_HUMAN	IRS1_HUMAN
TNR1A_HUMAN	JAK1_HUMAN
NARG2_HUMAN	JAK2_HUMAN
NEMO_HUMAN	JAK2_HUMAN
TNR1A_HUMAN	JAK2_HUMAN
2ABB_HUMAN	JUN_HUMAN
ANDR_HUMAN	JUN_HUMAN
BRCA1_HUMAN	JUN_HUMAN
CDN1A_HUMAN	JUN_HUMAN
CSN5_HUMAN	JUN_HUMAN
ESR1_HUMAN	JUN_HUMAN
FOS_HUMAN	JUN_HUMAN
GSK3B_HUMAN	JUN_HUMAN
HSP7C_HUMAN	JUN_HUMAN
MK01_HUMAN	JUN_HUMAN
MK03_HUMAN	JUN_HUMAN
MK14_HUMAN	JUN_HUMAN
NARG2_HUMAN	JUN_HUMAN
PAG1_HUMAN	JUN_HUMAN
PRKDC_HUMAN	JUN_HUMAN
RS27A_HUMAN	JUN_HUMAN
UBB_HUMAN	JUN_HUMAN

Table S1. *Cont.*

Protein 1	Protein 2
VAV_HUMAN	JUN_HUMAN
1433Z_HUMAN	KAPCA_HUMAN
BCL2_HUMAN	KAPCA_HUMAN
ESR1_HUMAN	KAPCA_HUMAN
1433Z_HUMAN	KPCA_HUMAN
EZRI_HUMAN	KPCA_HUMAN
PRKDC_HUMAN	KPCD_HUMAN
CASP3_HUMAN	KPCZ_HUMAN
CASP6_HUMAN	KPCZ_HUMAN
CASP7_HUMAN	KPCZ_HUMAN
CASP8_HUMAN	KPCZ_HUMAN
GCR_HUMAN	LCK_HUMAN
HS90A_HUMAN	LCK_HUMAN
CASP3_HUMAN	LYN_HUMAN
1433E_HUMAN	M3K1_HUMAN
ARRB1_HUMAN	M3K1_HUMAN
ARRB2_HUMAN	M3K1_HUMAN
HS90A_HUMAN	M3K1_HUMAN
RS27A_HUMAN	M3K1_HUMAN
TRAF2_HUMAN	M3K1_HUMAN
UB2D1_HUMAN	M3K1_HUMAN
UB2D2_HUMAN	M3K1_HUMAN
UBC_HUMAN	M3K1_HUMAN
UBC9_HUMAN	M3K1_HUMAN
CASP3_HUMAN	M3K14_HUMAN
CASPA_HUMAN	M3K14_HUMAN
IKKB_HUMAN	M3K14_HUMAN
NEMO_HUMAN	M3K14_HUMAN
A4_HUMAN	M3K5_HUMAN
ARRB1_HUMAN	M3K5_HUMAN
ARRB2_HUMAN	M3K5_HUMAN
HSP71_HUMAN	M3K5_HUMAN
TRAF2_HUMAN	M3K5_HUMAN
RS27A_HUMAN	M3K7_HUMAN
UBC_HUMAN	M3K7_HUMAN
B2CL1_HUMAN	MCL1_HUMAN
BAK_HUMAN	MCL1_HUMAN
BAX_HUMAN	MCL1_HUMAN
BCL10_HUMAN	MCL1_HUMAN
BIRC2_HUMAN	MCL1_HUMAN
CASP1_HUMAN	MCL1_HUMAN
CASP2_HUMAN	MCL1_HUMAN
GSK3B_HUMAN	MCL1_HUMAN
MK01_HUMAN	MCL1_HUMAN
PCNA_HUMAN	MCL1_HUMAN
RIPK2_HUMAN	MCL1_HUMAN
EP300_HUMAN	MDM2_HUMAN
ESR1_HUMAN	MDM2_HUMAN
P53_HUMAN	MDM2_HUMAN
P73_HUMAN	MDM2_HUMAN
CASP8_HUMAN	MK01_HUMAN
CRKL_HUMAN	MK01_HUMAN
ESR1_HUMAN	MK01_HUMAN
FOS_HUMAN	MK01_HUMAN
G3P_HUMAN	MK01_HUMAN
PTN1_HUMAN	MK01_HUMAN

Table S1. *Cont.*

Protein 1	Protein 2
SYUA_HUMAN	MK01_HUMAN
BCL2_HUMAN	MK03_HUMAN
CASP8_HUMAN	MK03_HUMAN
CEBPB_HUMAN	MK03_HUMAN
ESR1_HUMAN	MK03_HUMAN
MK01_HUMAN	MK03_HUMAN
SYUA_HUMAN	MK03_HUMAN
CD2A2_HUMAN	MK08_HUMAN
EZRI_HUMAN	MK08_HUMAN
JUN_HUMAN	MK08_HUMAN
SMAD3_HUMAN	MK08_HUMAN
TRAF6_HUMAN	MK08_HUMAN
BCL2_HUMAN	MK14_HUMAN
CFLAR_HUMAN	MP2K1_HUMAN
RAF1_HUMAN	MP2K1_HUMAN
TRAF3_HUMAN	MP2K1_HUMAN
ABL1_HUMAN	MUC1_HUMAN
ESR1_HUMAN	MUC1_HUMAN
GSK3B_HUMAN	MUC1_HUMAN
HDAC1_HUMAN	MUC1_HUMAN
JUN_HUMAN	MUC1_HUMAN
LCK_HUMAN	MUC1_HUMAN
RXRA_HUMAN	MUC1_HUMAN
BRCA1_HUMAN	MYC_HUMAN
CSK21_HUMAN	MYC_HUMAN
MK03_HUMAN	MYC_HUMAN
MK08_HUMAN	MYC_HUMAN
NARG2_HUMAN	MYC_HUMAN
RUVB2_HUMAN	MYC_HUMAN
SET_HUMAN	MYC_HUMAN
TBA3C_HUMAN	MYC_HUMAN
TBA4A_HUMAN	MYC_HUMAN
UBB_HUMAN	MYC_HUMAN
UBC_HUMAN	MYC_HUMAN
ABL1_HUMAN	NARG2_HUMAN
EP300_HUMAN	NARG2_HUMAN
PRKDC_HUMAN	NARG2_HUMAN
PML_HUMAN	NCOR1_HUMAN
ZBT16_HUMAN	NCOR1_HUMAN
BCL6_HUMAN	NCOR2_HUMAN
JUN_HUMAN	NCOR2_HUMAN
STA5B_HUMAN	NCOR2_HUMAN
ZBT16_HUMAN	NCOR2_HUMAN
HS90A_HUMAN	NEMO_HUMAN
HSP74_HUMAN	NEMO_HUMAN
IKBA_HUMAN	NEMO_HUMAN
IKKA_HUMAN	NEMO_HUMAN
M3K3_HUMAN	NEMO_HUMAN
RIP_HUMAN	NEMO_HUMAN
UBB_HUMAN	NEMO_HUMAN
UBC_HUMAN	NEMO_HUMAN
ANDR_HUMAN	NFKB1_HUMAN
HSP74_HUMAN	NFKB1_HUMAN
IKKA_HUMAN	NFKB1_HUMAN
NEMO_HUMAN	NFKB1_HUMAN
NFKB2_HUMAN	NFKB1_HUMAN

Table S1. *Cont.*

Protein 1	Protein 2
TF65_HUMAN	NFKB1_HUMAN
IKKB_HUMAN	NFKB2_HUMAN
NEMO_HUMAN	NFKB2_HUMAN
TF65_HUMAN	NFKB2_HUMAN
HIF1A_HUMAN	NOTC1_HUMAN
AKT1_HUMAN	NR4A1_HUMAN
MK08_HUMAN	NR4A1_HUMAN
CAV1_HUMAN	NTRK1_HUMAN
CDK2_HUMAN	NTRK1_HUMAN
CDK5_HUMAN	NTRK1_HUMAN
CRCM_HUMAN	NTRK1_HUMAN
CRKL_HUMAN	NTRK1_HUMAN
MK01_HUMAN	NTRK1_HUMAN
MK14_HUMAN	NTRK1_HUMAN
SRC_HUMAN	NTRK1_HUMAN
TNR16_HUMAN	NTRK1_HUMAN
UBB_HUMAN	NTRK1_HUMAN
BAD_HUMAN	ORN_HUMAN
P53_HUMAN	ORN_HUMAN
1433Z_HUMAN	P53_HUMAN
BRCA1_HUMAN	P53_HUMAN
CD2A2_HUMAN	P53_HUMAN
CDK1_HUMAN	P53_HUMAN
CSK21_HUMAN	P53_HUMAN
CSN5_HUMAN	P53_HUMAN
EP300_HUMAN	P53_HUMAN
GRP75_HUMAN	P53_HUMAN
IKKA_HUMAN	P53_HUMAN
MUC1_HUMAN	P53_HUMAN
NARG2_HUMAN	P53_HUMAN
NR4A1_HUMAN	P53_HUMAN
PAG1_HUMAN	P53_HUMAN
SUMO1_HUMAN	P53_HUMAN
UBC_HUMAN	P53_HUMAN
UBC9_HUMAN	P53_HUMAN
UBP7_HUMAN	P53_HUMAN
PAG1_HUMAN	P73_HUMAN
SUMO1_HUMAN	P73_HUMAN
ESR1_HUMAN	P85A_HUMAN
ABL1_HUMAN	PAG1_HUMAN
CTNB1_HUMAN	PAG1_HUMAN
ESR1_HUMAN	PAG1_HUMAN
SLK_HUMAN	PAG1_HUMAN
VAV_HUMAN	PAG1_HUMAN
ACTB_HUMAN	PAK2_HUMAN
CASP3_HUMAN	PAK2_HUMAN
MYC_HUMAN	PAK2_HUMAN
RB_HUMAN	PAK2_HUMAN
CASP3_HUMAN	PARP1_HUMAN
CDN1A_HUMAN	PARP1_HUMAN
NEMO_HUMAN	PARP1_HUMAN
P53_HUMAN	PARP1_HUMAN
RXRA_HUMAN	PARP1_HUMAN
CASP3_HUMAN	PAXI_HUMAN
PP2AA_HUMAN	PAXI_HUMAN
PTPRC_HUMAN	PAXI_HUMAN

Table S1.*Cont.*

Protein 1	Protein 2
IKKE_HUMAN	PCNA_HUMAN
TF65_HUMAN	PCNA_HUMAN
RASA1_HUMAN	PGFRB_HUMAN
MYC_HUMAN	PIN1_HUMAN
PAK2_HUMAN	PIN1_HUMAN
TF65_HUMAN	PIN1_HUMAN
VHL_HUMAN	PIN1_HUMAN
CASP3_HUMAN	PKN1_HUMAN
MK14_HUMAN	PML_HUMAN
B2CL1_HUMAN	PP1A_HUMAN
TGFR1_HUMAN	PP1A_HUMAN
2ABB_HUMAN	PP2AA_HUMAN
MK03_HUMAN	PP2AA_HUMAN
MYC_HUMAN	PP2AA_HUMAN
CASP2_HUMAN	PRKDC_HUMAN
CASP3_HUMAN	PRKDC_HUMAN
CFTR_HUMAN	PRKDC_HUMAN
GRB2_HUMAN	PRKDC_HUMAN
H2AX_HUMAN	PRKDC_HUMAN
M3K3_HUMAN	PRKDC_HUMAN
MYC_HUMAN	PRKDC_HUMAN
NEMO_HUMAN	PRKDC_HUMAN
NFKB1_HUMAN	PRKDC_HUMAN
NFKB2_HUMAN	PRKDC_HUMAN
SP1_HUMAN	PRKDC_HUMAN
TF65_HUMAN	PRKDC_HUMAN
TNR1A_HUMAN	PRKDC_HUMAN
TNR1B_HUMAN	PRKDC_HUMAN
TRADD_HUMAN	PRKDC_HUMAN
CDK5_HUMAN	PSN1_HUMAN
KAPCA_HUMAN	PSN1_HUMAN
P85A_HUMAN	PSN1_HUMAN
CSK21_HUMAN	PSN2_HUMAN
CDK1_HUMAN	PTN1_HUMAN
TNR1A_HUMAN	PTN11_HUMAN
PAG1_HUMAN	PTN6_HUMAN
SMAD3_HUMAN	PTN6_HUMAN
TNR6_HUMAN	PTN6_HUMAN
1433Z_HUMAN	PYR1_HUMAN
2ABB_HUMAN	PYR1_HUMAN
NEMO_HUMAN	PYR1_HUMAN
TNR1A_HUMAN	PYR1_HUMAN
TNR1B_HUMAN	PYR1_HUMAN
TRADD_HUMAN	PYR1_HUMAN
CASP7_HUMAN	RAC1_HUMAN
1433B_HUMAN	RAF1_HUMAN
1433Z_HUMAN	RAF1_HUMAN
BCL2_HUMAN	RAF1_HUMAN
CH60_HUMAN	RAF1_HUMAN
GCR_HUMAN	RAF1_HUMAN
ORN_HUMAN	RAF1_HUMAN
RASH_HUMAN	RAF1_HUMAN
UB2D2_HUMAN	RAF1_HUMAN
UBC_HUMAN	RAF1_HUMAN
CASP3_HUMAN	RASA1_HUMAN
CASP7_HUMAN	RASA1_HUMAN

Table S1. *Cont.*

Protein 1	Protein 2
RASH_HUMAN	RASA1_HUMAN
CRKL_HUMAN	RASH_HUMAN
MK03_HUMAN	RASH_HUMAN
MK08_HUMAN	RASH_HUMAN
RAF1_HUMAN	RASH_HUMAN
CEBPB_HUMAN	RB_HUMAN
HDAC1_HUMAN	RB_HUMAN
HSP71_HUMAN	RHOA_HUMAN
TNR6_HUMAN	RHOA_HUMAN
HSPB1_HUMAN	RIF1_HUMAN
TNR6_HUMAN	RIF1_HUMAN
CASPA_HUMAN	RIP_HUMAN
CFLAR_HUMAN	RIP_HUMAN
EGFR_HUMAN	RIP_HUMAN
TNR6_HUMAN	RIP_HUMAN
TR10B_HUMAN	RIP_HUMAN
TRADD_HUMAN	RIP_HUMAN
TRAF1_HUMAN	RIP_HUMAN
TRAF3_HUMAN	RIP_HUMAN
TNR1A_HUMAN	RIPK1_HUMAN
TNR6_HUMAN	RIPK1_HUMAN
TRADD_HUMAN	RIPK1_HUMAN
NEMO_HUMAN	RIPK2_HUMAN
1433Z_HUMAN	RS27A_HUMAN
2ABB_HUMAN	RS27A_HUMAN
BIRC2_HUMAN	RS27A_HUMAN
CDN1A_HUMAN	RS27A_HUMAN
DAXX_HUMAN	RS27A_HUMAN
IKBA_HUMAN	RS27A_HUMAN
IKKE_HUMAN	RS27A_HUMAN
IRAK1_HUMAN	RS27A_HUMAN
MYC_HUMAN	RS27A_HUMAN
NEMO_HUMAN	RS27A_HUMAN
P53_HUMAN	RS27A_HUMAN
P73_HUMAN	RS27A_HUMAN
RHOA_HUMAN	RS27A_HUMAN
TERA_HUMAN	RS27A_HUMAN
TGFR1_HUMAN	RS27A_HUMAN
TNR1A_HUMAN	RS27A_HUMAN
TNR1B_HUMAN	RS27A_HUMAN
TRADD_HUMAN	RS27A_HUMAN
TRAF1_HUMAN	RS27A_HUMAN
XIAP_HUMAN	RS27A_HUMAN
1433Z_HUMAN	RUVB2_HUMAN
IKKE_HUMAN	RUVB2_HUMAN
M3K1_HUMAN	RUVB2_HUMAN
M3K7_HUMAN	RUVB2_HUMAN
TRAF2_HUMAN	RUVB2_HUMAN
TRAF6_HUMAN	RUVB2_HUMAN
1433Z_HUMAN	SET_HUMAN
IKKE_HUMAN	SET_HUMAN
NEMO_HUMAN	SET_HUMAN
RAC1_HUMAN	SET_HUMAN
VHL_HUMAN	SET_HUMAN
EGFR_HUMAN	SHC1_HUMAN
MYC_HUMAN	SIN3A_HUMAN

Table S1.*Cont.*

Protein 1	Protein 2
NFKB1_HUMAN	SIN3A_HUMAN
SUMO1_HUMAN	SIN3A_HUMAN
TNFL6_HUMAN	SLK_HUMAN
GSK3B_HUMAN	SMAD1_HUMAN
RASA1_HUMAN	SMAD2_HUMAN
RHOA_HUMAN	SMAD2_HUMAN
GSK3B_HUMAN	SMAD3_HUMAN
MK14_HUMAN	SMAD3_HUMAN
UBC_HUMAN	SMAD3_HUMAN
CEBPB_HUMAN	SMAD4_HUMAN
TNR1B_HUMAN	SMUF2_HUMAN
ABL1_HUMAN	SP1_HUMAN
BRCA1_HUMAN	SP1_HUMAN
CASP3_HUMAN	SP1_HUMAN
ESR1_HUMAN	SP1_HUMAN
SIN3A_HUMAN	SP1_HUMAN
NTRK1_HUMAN	SQSTM_HUMAN
RS27A_HUMAN	SQSTM_HUMAN
SMAD1_HUMAN	SQSTM_HUMAN
TR10A_HUMAN	SQSTM_HUMAN
UBB_HUMAN	SQSTM_HUMAN
UBC_HUMAN	SQSTM_HUMAN
MK01_HUMAN	SRC_HUMAN
NR4A1_HUMAN	SRC_HUMAN
TNR6_HUMAN	SRC_HUMAN
TRAF1_HUMAN	SRC_HUMAN
TRAF3_HUMAN	SRC_HUMAN
ESR1_HUMAN	STA5A_HUMAN
PGFRB_HUMAN	STA5A_HUMAN
FOS_HUMAN	STAT1_HUMAN
NARG2_HUMAN	STAT1_HUMAN
PAG1_HUMAN	STAT1_HUMAN
RAC1_HUMAN	STAT1_HUMAN
SMAD4_HUMAN	STAT1_HUMAN
TRADD_HUMAN	STAT1_HUMAN
ESR1_HUMAN	STAT3_HUMAN
NEMO_HUMAN	SUMO1_HUMAN
ZBT16_HUMAN	SUMO1_HUMAN
1433E_HUMAN	SYUA_HUMAN
CSK21_HUMAN	SYUA_HUMAN
FYN_HUMAN	SYUA_HUMAN
KSYK_HUMAN	SYUA_HUMAN
SLK_HUMAN	SYUA_HUMAN
UBC_HUMAN	SYUA_HUMAN
VIME_HUMAN	SYUA_HUMAN
1433Z_HUMAN	TAU_HUMAN
CASP1_HUMAN	TAU_HUMAN
CASP3_HUMAN	TAU_HUMAN
CASP6_HUMAN	TAU_HUMAN
CASP7_HUMAN	TAU_HUMAN
CASP8_HUMAN	TAU_HUMAN
IKBA_HUMAN	TBA3C_HUMAN
IKKB_HUMAN	TBA3C_HUMAN
IKKE_HUMAN	TBA3C_HUMAN
M3K1_HUMAN	TBA3C_HUMAN
M3K7_HUMAN	TBA3C_HUMAN

Table S1. *Cont.*

Protein 1	Protein 2
NEMO_HUMAN	TBA3C_HUMAN
NFKB1_HUMAN	TBA3C_HUMAN
RIPK1_HUMAN	TBA3C_HUMAN
RIPK2_HUMAN	TBA3C_HUMAN
SLK_HUMAN	TBA3C_HUMAN
TF65_HUMAN	TBA3C_HUMAN
TNR1A_HUMAN	TBA3C_HUMAN
TNR1B_HUMAN	TBA3C_HUMAN
TRADD_HUMAN	TBA3C_HUMAN
BRCA1_HUMAN	TBA4A_HUMAN
EGFR_HUMAN	TBA4A_HUMAN
RAC1_HUMAN	TBA4A_HUMAN
IKKB_HUMAN	TBB4_HUMAN
IKKE_HUMAN	TBB4_HUMAN
M3K1_HUMAN	TBB4_HUMAN
M3K7_HUMAN	TBB4_HUMAN
NEMO_HUMAN	TBB4_HUMAN
NFKB1_HUMAN	TBB4_HUMAN
NR4A1_HUMAN	TBB4_HUMAN
RIPK1_HUMAN	TBB4_HUMAN
RIPK2_HUMAN	TBB4_HUMAN
TF65_HUMAN	TBB4_HUMAN
TNR1A_HUMAN	TBB4_HUMAN
TNR1B_HUMAN	TBB4_HUMAN
TRADD_HUMAN	TBB4_HUMAN
1433B_HUMAN	TBB5_HUMAN
1433G_HUMAN	TBB5_HUMAN
1433Z_HUMAN	TBB5_HUMAN
2ABB_HUMAN	TBB5_HUMAN
ARRB1_HUMAN	TBB5_HUMAN
EGFR_HUMAN	TBB5_HUMAN
GRB2_HUMAN	TBB5_HUMAN
IKBA_HUMAN	TBB5_HUMAN
IKKA_HUMAN	TBB5_HUMAN
IKKB_HUMAN	TBB5_HUMAN
IKKE_HUMAN	TBB5_HUMAN
JUN_HUMAN	TBB5_HUMAN
M3K1_HUMAN	TBB5_HUMAN
M3K14_HUMAN	TBB5_HUMAN
M3K3_HUMAN	TBB5_HUMAN
M3K7_HUMAN	TBB5_HUMAN
NEMO_HUMAN	TBB5_HUMAN
NFKB1_HUMAN	TBB5_HUMAN
NFKB2_HUMAN	TBB5_HUMAN
RIPK1_HUMAN	TBB5_HUMAN
RIPK2_HUMAN	TBB5_HUMAN
TBA4A_HUMAN	TBB5_HUMAN
TBB4_HUMAN	TBB5_HUMAN
TF65_HUMAN	TBB5_HUMAN
TNR1A_HUMAN	TBB5_HUMAN
TNR1B_HUMAN	TBB5_HUMAN
TRADD_HUMAN	TBB5_HUMAN
1433Z_HUMAN	TERA_HUMAN
1B42_HUMAN	TERA_HUMAN
HSP74_HUMAN	TERA_HUMAN
NARG2_HUMAN	TERA_HUMAN

Table S1.*Cont.*

Protein 1	Protein 2
ANDR_HUMAN	TF65_HUMAN
CSK21_HUMAN	TF65_HUMAN
ESR1_HUMAN	TF65_HUMAN
HSP74_HUMAN	TF65_HUMAN
IKBA_HUMAN	TF65_HUMAN
KAPCA_HUMAN	TF65_HUMAN
NARG2_HUMAN	TF65_HUMAN
PAG1_HUMAN	TF65_HUMAN
UBC_HUMAN	TF65_HUMAN
1433Z_HUMAN	TGFR1_HUMAN
TGFR2_HUMAN	TGFR1_HUMAN
TNR1A_HUMAN	TNFA_HUMAN
CASPA_HUMAN	TNFL6_HUMAN
DAXX_HUMAN	TNFL6_HUMAN
EZRI_HUMAN	TNFL6_HUMAN
FYN_HUMAN	TNFL6_HUMAN
NCK1_HUMAN	TNFL6_HUMAN
SUMO1_HUMAN	TNFL6_HUMAN
TNR6_HUMAN	TNFL6_HUMAN
CASPA_HUMAN	TNR16_HUMAN
NTRK1_HUMAN	TNR16_HUMAN
SHC1_HUMAN	TNR16_HUMAN
TNR6_HUMAN	TNR16_HUMAN
CFLAR_HUMAN	TNR1A_HUMAN
EZRI_HUMAN	TNR1A_HUMAN
FADD_HUMAN	TNR1A_HUMAN
RIP_HUMAN	TNR1A_HUMAN
RIPK1_HUMAN	TNR1A_HUMAN
TNFA_HUMAN	TNR1A_HUMAN
TRADD_HUMAN	TNR1A_HUMAN
STAT1_HUMAN	TNR1B_HUMAN
TNFA_HUMAN	TNR1B_HUMAN
EWS_HUMAN	TNR6_HUMAN
FADD_HUMAN	TNR6_HUMAN
FYN_HUMAN	TNR6_HUMAN
TNFL6_HUMAN	TNR6_HUMAN
BTK_HUMAN	TR10A_HUMAN
EZRI_HUMAN	TR10B_HUMAN
RIPK1_HUMAN	TRADD_HUMAN
TNR1A_HUMAN	TRADD_HUMAN
CASP3_HUMAN	TRAF1_HUMAN
CASP8_HUMAN	TRAF1_HUMAN
M3K14_HUMAN	TRAF1_HUMAN
IKKA_HUMAN	TRAF2_HUMAN
IKKE_HUMAN	TRAF2_HUMAN
M3K14_HUMAN	TRAF2_HUMAN
NEMO_HUMAN	TRAF2_HUMAN
RIPK2_HUMAN	TRAF2_HUMAN
TNR1B_HUMAN	TRAF2_HUMAN
TRADD_HUMAN	TRAF2_HUMAN
TRAF1_HUMAN	TRAF2_HUMAN
CASP3_HUMAN	TRAF3_HUMAN
M3K14_HUMAN	TRAF3_HUMAN
BMR1B_HUMAN	TRAF6_HUMAN
FHL2_HUMAN	TRAF6_HUMAN
CASP8_HUMAN	UB2D1_HUMAN

Table S1. *Cont.*

Protein 1	Protein 2
IKBA_HUMAN	UB2D1_HUMAN
CTNB1_HUMAN	UB2D2_HUMAN
IKBA_HUMAN	UB2D2_HUMAN
BRCA1_HUMAN	UBB_HUMAN
EGFR_HUMAN	UBB_HUMAN
P53_HUMAN	UBB_HUMAN
SYUA_HUMAN	UBB_HUMAN
TGFR1_HUMAN	UBB_HUMAN
TRAF6_HUMAN	UBB_HUMAN
XIAP_HUMAN	UBB_HUMAN
1433Z_HUMAN	UBC_HUMAN
AKT1_HUMAN	UBC_HUMAN
BAX_HUMAN	UBC_HUMAN
BCL10_HUMAN	UBC_HUMAN
BCL2_HUMAN	UBC_HUMAN
BIRC2_HUMAN	UBC_HUMAN
CASP8_HUMAN	UBC_HUMAN
CDN1A_HUMAN	UBC_HUMAN
CTNB1_HUMAN	UBC_HUMAN
DAXX_HUMAN	UBC_HUMAN
EGFR_HUMAN	UBC_HUMAN
ERBB2_HUMAN	UBC_HUMAN
ERBB3_HUMAN	UBC_HUMAN
ESR1_HUMAN	UBC_HUMAN
IKKE_HUMAN	UBC_HUMAN
JUN_HUMAN	UBC_HUMAN
M3K5_HUMAN	UBC_HUMAN
MCL1_HUMAN	UBC_HUMAN
MK01_HUMAN	UBC_HUMAN
NOTC1_HUMAN	UBC_HUMAN
P73_HUMAN	UBC_HUMAN
PML_HUMAN	UBC_HUMAN
PSN2_HUMAN	UBC_HUMAN
RHOA_HUMAN	UBC_HUMAN
TERA_HUMAN	UBC_HUMAN
TNFL6_HUMAN	UBC_HUMAN
TNR1A_HUMAN	UBC_HUMAN
TNR1B_HUMAN	UBC_HUMAN
TRADD_HUMAN	UBC_HUMAN
TRAF1_HUMAN	UBC_HUMAN
TRAF2_HUMAN	UBC_HUMAN
TRAF3_HUMAN	UBC_HUMAN
TRAF6_HUMAN	UBC_HUMAN
XIAP_HUMAN	UBC_HUMAN
ZBT16_HUMAN	UBC_HUMAN
ESR1_HUMAN	UBC9_HUMAN
IKKE_HUMAN	UBC9_HUMAN
JUN_HUMAN	UBC9_HUMAN
TNR6_HUMAN	UBC9_HUMAN
P53_HUMAN	UBP7_HUMAN
SMAD3_HUMAN	UBP7_HUMAN
CBL_HUMAN	VAV_HUMAN
HDAC2_HUMAN	VHL_HUMAN
NR4A1_HUMAN	VHL_HUMAN
SP1_HUMAN	VHL_HUMAN
1433Z_HUMAN	VIME_HUMAN

Table S1.*Cont.*

Protein 1	Protein 2
2ABB_HUMAN	VIME_HUMAN
CDK1_HUMAN	VIME_HUMAN
M3K1_HUMAN	VIME_HUMAN
NFKB1_HUMAN	VIME_HUMAN
RIPK2_HUMAN	VIME_HUMAN
TNR1B_HUMAN	VIME_HUMAN
TRADD_HUMAN	VIME_HUMAN
AKT1_HUMAN	XIAP_HUMAN
M3K7_HUMAN	XIAP_HUMAN
NOTC1_HUMAN	XIAP_HUMAN
1433Z_HUMAN	XPO1_HUMAN
ABL1_HUMAN	XPO1_HUMAN
HDAC3_HUMAN	XPO1_HUMAN
TNR1B_HUMAN	XPO1_HUMAN
1433Z_HUMAN	XRCC6_HUMAN
MK08_HUMAN	XRCC6_HUMAN
MYC_HUMAN	XRCC6_HUMAN
PRKDC_HUMAN	XRCC6_HUMAN
TERA_HUMAN	XRCC6_HUMAN
VAV_HUMAN	XRCC6_HUMAN
EF1A1_HUMAN	ZBT16_HUMAN
M3K3_HUMAN	ZBT16_HUMAN

Table S2. Known apoptotic gene involved in plant lectin-induced cancer cell death.

Gene Name	Protein Name
CASP8/MCH5	Caspase-8
CASP6/MCH2	Caspase-6
CASP7/MCH3	Caspase-7
CASP3/CPP32	Caspase-3
CASP10/MCH4	Caspase-10
CASP9/MCH6	Caspase-9
TP53/P53	Cellular tumor antigen p53
TP73/P73	Tumor protein p73
BAD/BBC6/BCL2L8	Bcl2 antagonist of cell death
BAX/BCL2L4	Apoptosis regulator BAX
CDKN1A/CAP20/CDKN1/CIP1/MDA6/PIC1/SDI1/WAF1	Cyclin-dependent kinase inhibitor 1
BCL2	Apoptosis regulator Bcl-2
BID	BH3-interacting domain death agonist
XIAP/API3/BIRC4/IAP3	E3 ubiquitin-protein ligase XIAP
TNFRSF1A/TNFAR/TNFR1	Tumor necrosis factor receptor superfamily member 1A
MAP2K1/MEK1/PRKMK1	Dual specificity mitogen-activated protein kinase kinase 1
RAF1/RAF	RAF proto-oncogene serine/threonine-protein kinase
YWHAZ	14-3-3 protein zeta/delta
CHUK/IKKA/TCF16	Inhibitor of nuclear factor κ-B kinase subunit α
BCL2L1/BCL2L/BCLX	Bcl-2-like protein 1
MAPK14/CSBP/CSBP1/CSBP2/CSPB1/MXI2/SAPK2A	Mitogen-activated protein kinase 14
RELA/NFKB3	Transcription factor p65
AKT1/PKB/RAC	RAC-α serine/threonine-protein kinase
RIPK1/RIP/RIP1	Receptor-interacting serine/threonine-protein kinase 1
RASA1/RASA	Ras GTPase-activating protein 1
PIK3R1/GRB1	Phosphatidylinositol 3-kinase regulatory subunit α
MAPK1/ERK2/PRKM1/PRKM2	Mitogen-activated protein kinase 1
CAD	CAD protein
PARP1/ADPRT/PPOL	Poly(ADP-ribose)polymerase 1

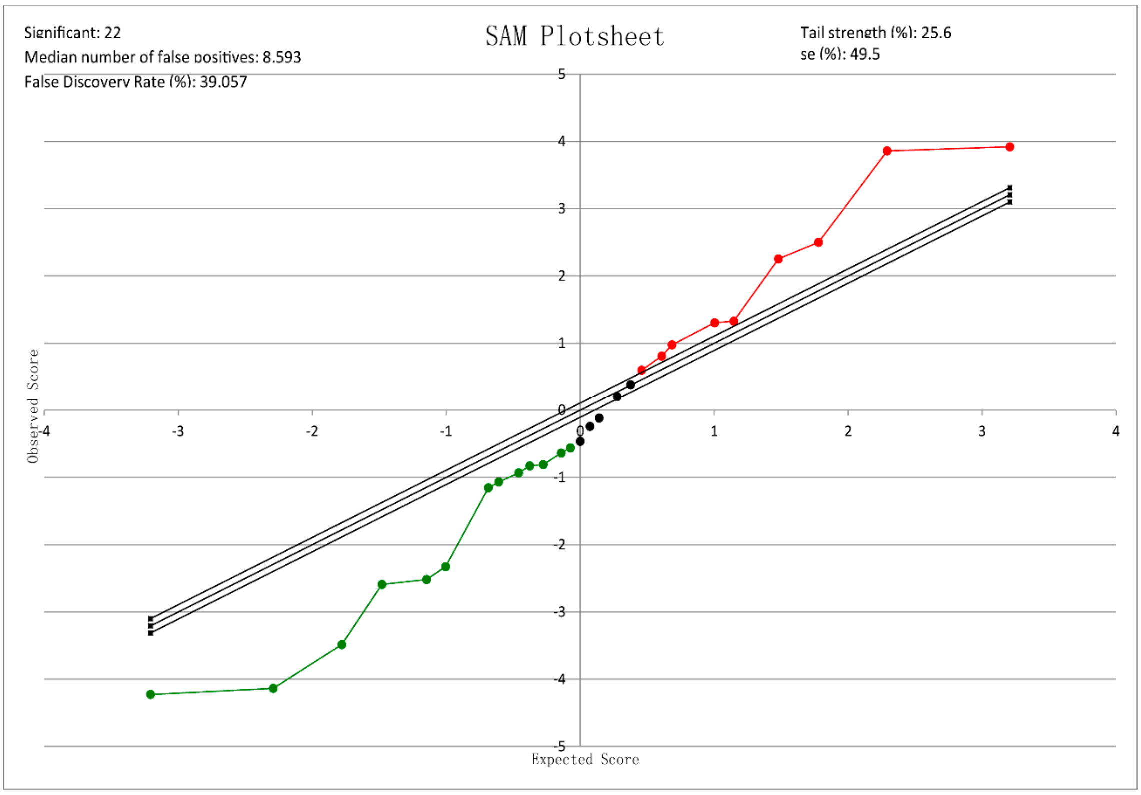

Figure S1. Evaluation of microarray data from mesothelioma. Green curve indicates negative significance down-regulated genes; red curve indicates positive significance up-regulated genes.
